# In vitro activity of phages against periprosthetic joint infection-associated staphylococcal biofilms

**DOI:** 10.1038/s41598-025-08759-9

**Published:** 2025-07-01

**Authors:** Krupa Parmar, Waqas Chaudhry, Joseph R. Fackler, John M. Sowers, Aravinda Vadlamudi, Kerryl Greenwood-Quaintance, Robin Patel

**Affiliations:** 1 Division of Clinical Microbiology, Department of Laboratory Medicine and Pathology, Mayo Clinic, 200 First Street SW, Rochester, MN 55905 USA; 2 Adaptive Phage Therapeutics Inc, Gaithersburg, MD USA; 3 Division of Public Health, Infectious Diseases, and Occupational Medicine, Department of Medicine, Mayo Clinic, Rochester, MN USA

**Keywords:** Phage susceptibility testing, Biofilms, *Staphylococcus aureus*, *Staphylococcus epidermidis*, *Staphylococcus lugdunensis*, Periprosthetic joint infection, Bacteriophages, Biofilms

## Abstract

Lytic phages are potential therapeutic options, based on their ability to lyse bacteria in vitro. Although many infection-types for which phage therapy is being considered involve biofilms, in vitro anti-biofilm activity of phage is poorly defined, in part due to a lack of standardized methods for assessment. Here, phages SaMD07phi1 and SaRBI05030phi5 were evaluated against *Staphylococcus aureus* SaMD07 and SaRBI05030, respectively, in biofilms formed in 96-well plates and on glass beads, and planktonically, in TSB and PBS, with endpoints including by CFUs and Biolog Omnilog hold times. The bead biofilm assay in TSB using the Omnilog (BBTO) was employed to test eight staphylococcal phages against *S. aureus*, *Staphylococcus epidermidis*, and *Staphylococcus lugdunensis* from periprosthetic joint infection. Biofilms on beads in TSB showed better eradication than in microtiter wells, with no significant changes with PBS in either format. CFU counts and Omnilog units correlated linearly through 8 h of testing. In the bead assay, CFU counts showed that phage SaMD07phi1 eliminated growth at 4 h, while SaRBI05030phi5 achieved a ~ 3-log reduction at 8 h; with Omnilog hold times of 37 and 28 h, respectively. Diverse activity and good reproducibility of the BBTO was observed among 8 phages, with SaMD07phi1 showing the highest activity. In conclusion the BBTO is a promising potential method for biofilm susceptibility testing.

## Introduction

Periprosthetic joint infections (PJIs) pose challenges due to the presence of biofilms, clusters of microorganisms adhered to surfaces and enclosed by a self-generated polymeric matrix^1^. Biofilm formation safeguards bacteria against environmental shifts and defense mechanisms, fostering resilience. Approximately 60–70% of hospital acquired infections are linked to biofilms on implanted devices^2^. Biofilms contribute to recurrence of infection^3^. Biofilms also present a challenge by conferring tolerance to many currently available antibiotics^4^. Together, these limitations have prompted an interest in phage therapy for managing biofilm-associated infections, including PJI^5–7^. The potential and limitations of phage-based treatments of PJI caused by *Staphylococcus* species has been described. For example, phage SEP1 has shown inefficient degradation of *S. epidermidis* biofilm due to the protective effect of the biofilm matrix^8^. In combination with antibiotics, phage Sb-1 has shown dose-dependent reductions in exopolysaccharide of methicillin-resistant *Staphylococcus aureus* biofilms and persister cells killing^9^. A prospective, open-label study with historical controls evaluating combined phage/antibiotic therapy of PJI showed a decrease in relapse of PJI compared to a control group receiving antibiotics alone^10^. A synergistic effect of phage Remus and vancomycin against MRSA biofilm-like aggregates in vitro and in vivo was reported^11^. Clinical studies have reported 33 bone and joint infection cases treated with phage therapy, where 87% achieved success, with the remaining relapsing with the same infecting microorganism or a different one^12^.

Limited studies have examined the activity of phages against biofilms in laboratory settings, including investigations into parameters of interest^13,14^. Several methods exist for studying biofilms in vitro, addressing structure, biomass, composition, and viability^15^. The ideal technique(s) to assess phage activity against biofilms remains to be defined^16^and should consider conditions of biofilm growth (e.g., bacterial characteristics, surface morphology and composition, biofilm age, growth medium, static or agitated incubation), phage traits (e.g., family/morphology, genome characteristics, burst size, latent period), treatment variables (e.g., phage buffer, phage titer, treatment duration, medium, static or agitated incubation), and interpretive criteria for assessing phage activity. Most studies assessing phage activity against biofilms have used biofilms formed for 24 h on surfaces of microtiter plates in either lysogeny broth (LB) or tryptic soy broth (TSB); most report the medium for phage treatment to be buffer or rich media, with the most common methods for evaluating biofilms involving quantification of biomass or bacterial cell numbers or (less commonly) measurement of metabolic activity^16^. As there are no established standards for evaluating phage activity against biofilms in microtiter plates, variability exists in parameters used for biofilm formation across studies. This variability has the potential to impact biofilm structure and, consequently, results of phage treatment. The diversity in methodologies employed highlights a need to establish standardized in vitro methods to facilitate characterization of phage-biofilm interactions and evaluation of phage activity to inform research and development of phage therapy for managing biofilm-associated infections.

In the present study, phage susceptibility was compared by assessment of biofilms on glass beads and in wells of microtiter plates, with two treatment media – tryptic soy broth (TSB) and phosphate buffered saline (PBS). Biofilm growth based on a Biolog Omnilog metabolic assay^17^ and quantification of viable cells by CFU counts were also compared.

## Methods

### Assessing experimental conditions for biofilm phage susceptibility testing

**Fig. 1:**
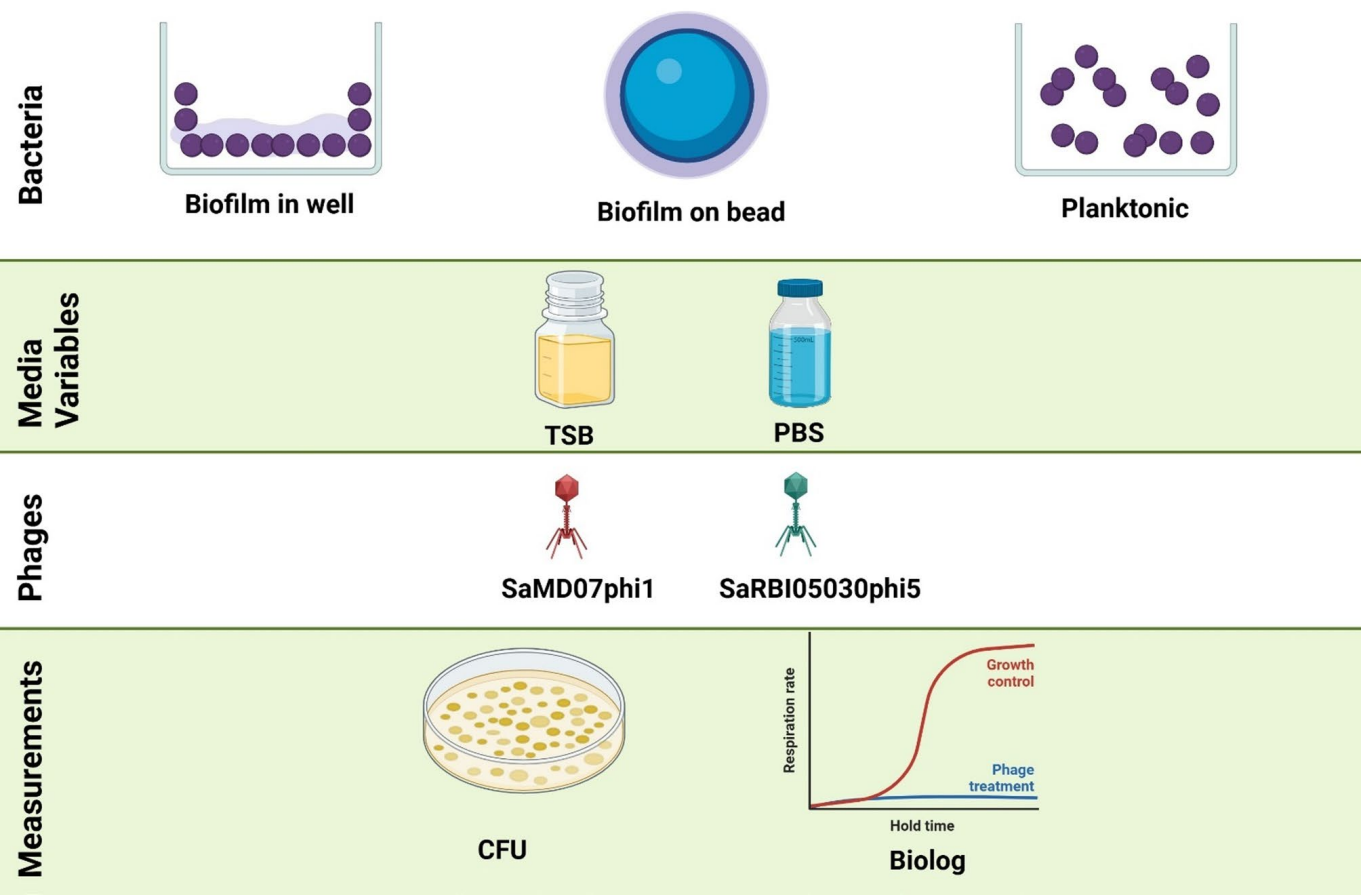
Biofilm phage susceptibility testing methods evaluated (created with BioRender.com).

Staphylococcal phages SaMD07phi1 and SaRBI05030phi5 were evaluated against biofilms of *S. aureus* SaMD07 and SaRBI05030, respectively. Biofilm growth was compared in parallel on two material surfaces – glasperlen 4 mm glass beads (Millipore-Sigma) and 96-well plates (351172; Falcon/Corning, USA) (Fig. 1). The starting inoculum was 10^5^ CFU/ml; biofilms were grown at 37 °C statically for 24 h (h), following which biofilms were gently washed in PBS thrice. Two media - phosphate buffered saline (PBS) and tryptic soy broth (TSB) - were compared in tandem for assessing phage susceptibility (Fig. 1). Four assay plates were prepared contemporaneously, that is, (a) biofilm on beads in PBS assay, (b) biofilm grown in 96-well plate in PBS, (c) biofilm on beads in TSB and (d) biofilm grown in 96-well plate in TSB. In assay plates (a) and (c), sterility controls for media were 100µL media and for phage were 25 µL phage (4 × 10^8^ PFU/mL) in 75 µL media, with a single sterile glass bead in each well. Biofilm controls contained biofilms grown on single beads, added to wells containing 100 µL medium. For plates (b) and (d), the same setup was used with biofilms grown on the well surfaces (instead of on beads). Biofilms were treated with 25 µL phage (4 × 10⁸ PFU/mL) in 75 µL medium. Plates (a) and (c) had biofilms on beads, while plates (b) and (d) had biofilms in the well, using the same treatment conditions. A similar setup was used to assess planktonic phage susceptibility for the purpose of comparison.

### Quantification of biofilms after treatment with phages

The effect of phage in controlling biofilm growth was monitored by assessing colony forming units (CFU) at 0, 4, 8, and 24 h. Plates for assessment of bacterial reduction were removed from the incubator at 0, 4, 8 and 24 h. For assay plates (b) and (d), biofilms were scraped from well surfaces using sterile toothpicks, vortexed for 30 s and serially diluted in PBS. Similar assessments of CFUs were performed for media and phage control wells. Dilutions were plated on to trypticase soy agar with 5% sheep blood (BD, Sparks, MD), incubated and colonies quantified. For assay plates (a) and (c), beads were placed in Eppendorf tubes containing 1 mL PBS, vortexed for 30 s, sonicated for 5 min (40 kHz, 0.22 W/cm^2^) and vortexed again for 30 s; sonicate fluid was quantitatively cultured as above, with results reported as CFU/mm^2^.

To compare colony reductions with a method using the Biolog Omnilog, similar assay plates were prepared for (a), (b), (c) and (d) and monitored every 15 min on the Omnilog over 48 h. The only difference was the use of Biolog-specific 96-well plates and the addition of 1% tetrazolium dye to TSB or PBS. The redox indicator dye measures changes in bacterial respiration via color change. Results were assessed as hold times – times through which bacterial growth is inhibited by phage as compared to controls calculated by subtracting the inflection times of phage treated biofilms from those of biofilm growth (positive control), ranging from 0 to 48 h. For example, if the inflection time for biofilm growth is 4 h, and the inflection time for phage-treated biofilm is 14 h, the hold time is 10 h.

### Testing activity of different phages using the established method

**Fig. 2:**
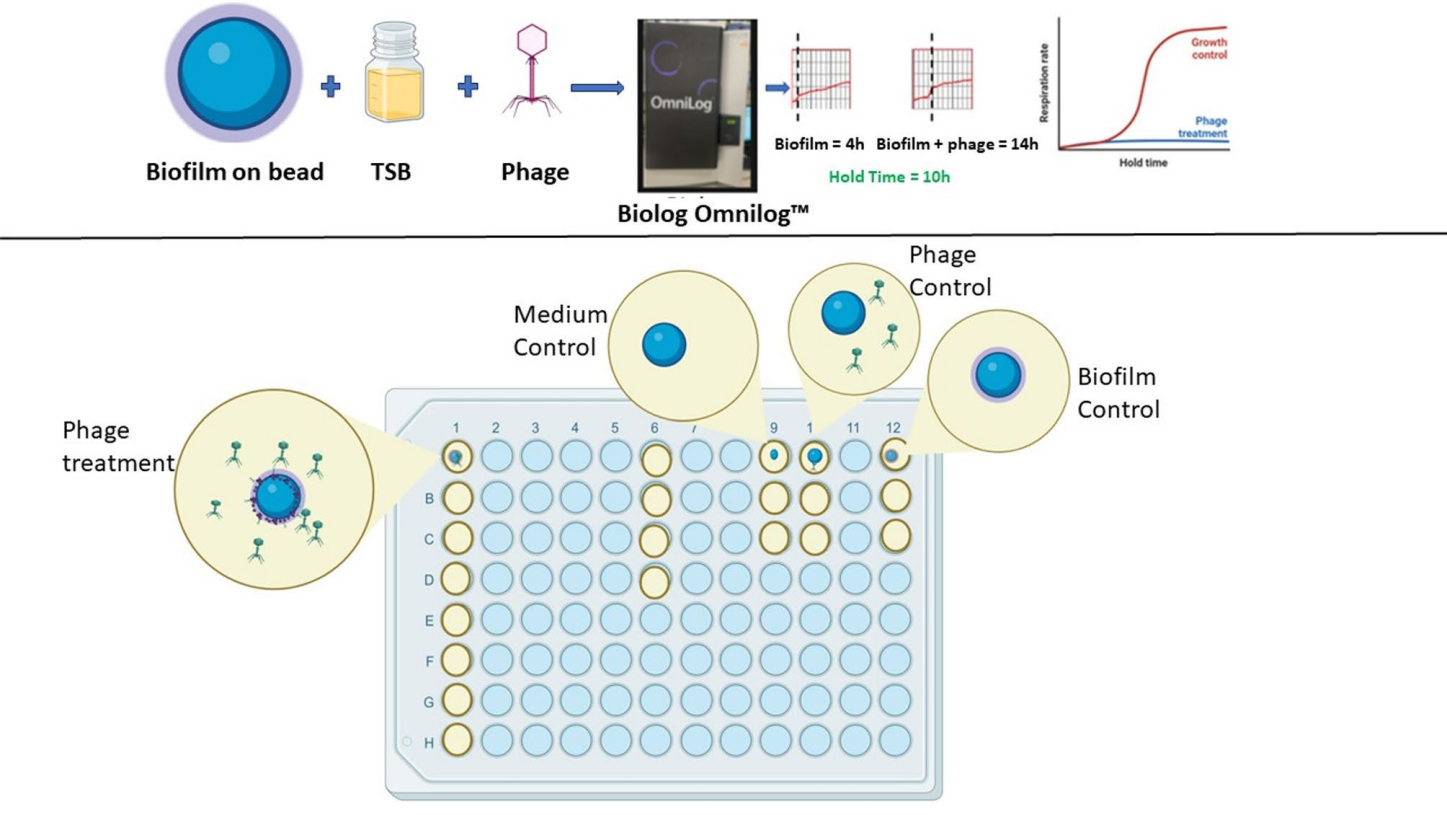
Representative figure for biofilm phage susceptibility testing using biofilms grown on beads assessed in TSB medium using the Biolog Omnilog method (created with BioRender.com).

Eight staphylococcal phages (SaMD07phi1, SaNSI1469phi1, SaWIQ0493phi1, SaRBI05030phi1, SaRBI03020phi1, Sl46407HNphi1, SaWIQ0488phi1 and SaMD22phi1[Adaptive Phage Therapeutics, Inc., USA]) were tested against biofilms of *S. aureus*, *S. epidermidis* and *S. lugdunensis* associated with PJI using the **b**ead **b**iofilm assay in **T**SB medium performed on the **O**mnilog (BBTO). Phages tested were lytic and lacked known toxin, virulence, antibiotic resistance, and integrase genes. Only phage-bacteria combinations for which planktonic phage activity was demonstrated using Biolog Omnilog were studied. Briefly, assay plates (Fig. 2) were prepared per plate (c) above, by growing biofilms on beads, using 1% tetrazolium dye in TSB as the assay medium, 25 µl phage (final concentration of 4 × 10^8^ PFU/ml) and monitored on the Biolog Omnilog instrument. Assay runs were performed in singlets on three different days. Phages were categorized based on mean hold times: those with hold times ≥ 8 hours were considered active, those with mean hold times below 4 h were considered inactive, and those with mean hold times between 4 and 8 hours were deemed to exhibit intermediate activity. Additionally, phages showing hold times ≥ 35 h were classified as highly active.

### Reproducibility of biofilm phage susceptibility method

Reproducibility of the biofilm phage susceptibility assay was estimated based on the mean hold time, standard deviation, and percent coefficient of variance (%CV) quantified over three replicate tests on three days. A %CV of ≤ 75% was considered as having valid reproducibility.

## Results

### Optimal experimental conditions for biofilm phage susceptibility testing

First, a comparison of two surfaces for biofilm growth − 4 mm glass beads and 96-well plates -, and two assay media – TSB and PBS – was performed, with two methods of quantifying activity, CFU quantification and the Biolog Omnilog assay. Bacterial counts of biofilms grown in 96-well plates ranged from 5.7 to 8.1 × 10^9^ CFU/mm^2^ for both SaMD07 and SaRBI05030, whereas biofilms grown on beads ranged from 2.0 to 2.8 × 10^7^ CFU/mm^2^ for both SaMD07 and SaRBI05030 at time zero (i.e., after 24 h of incubation). Assays were conducted in two media; quantities of SaMD07 and SaRBI05030 in PBS exhibited no change in either 96-well plates or on beads (data not shown). Consequently, additional testing was carried out in TSB.

**Fig. 3:**
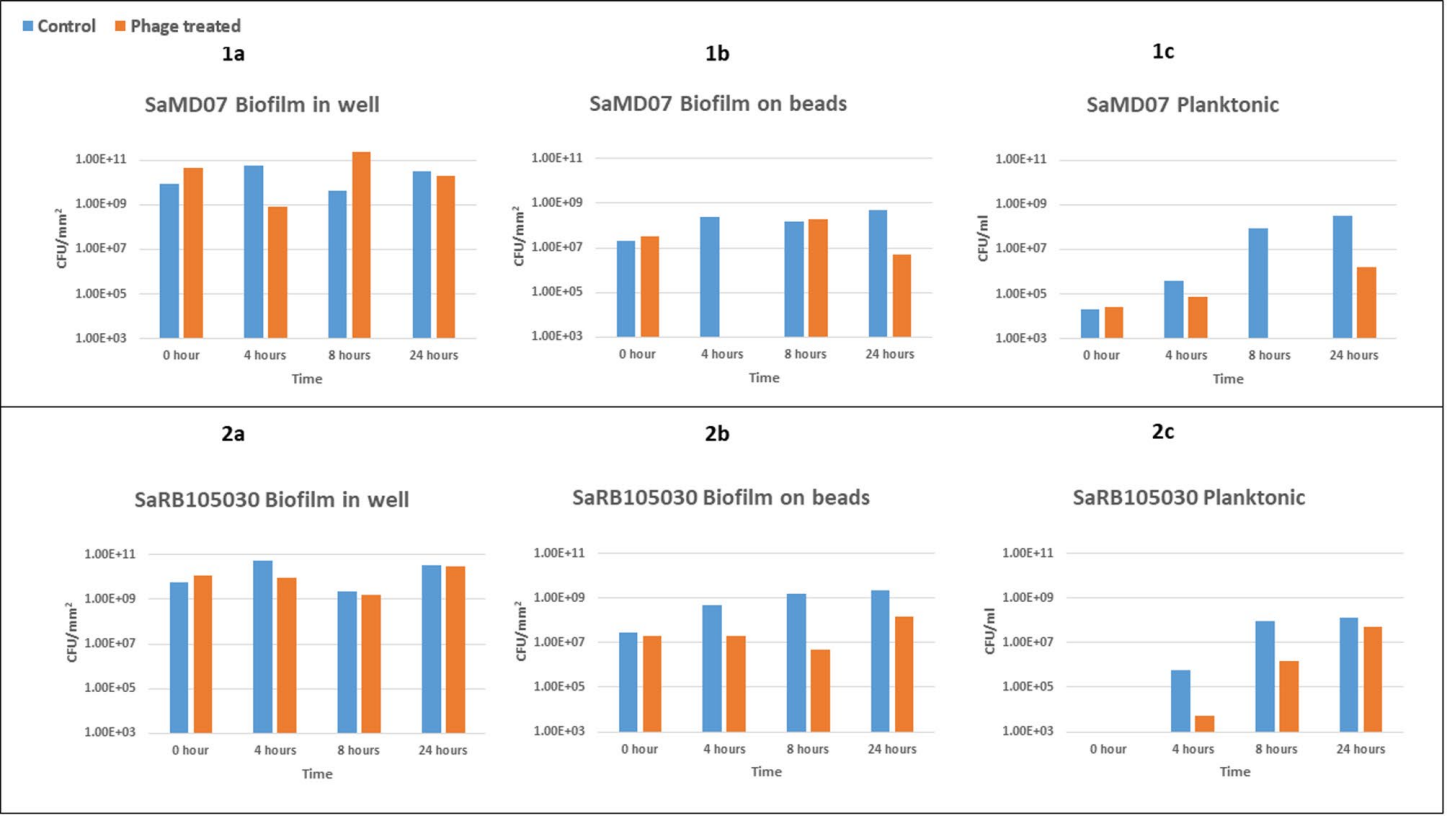
Results of quantitative culture of biofilms treated with phages SaMD07phi1 or SaRB105030phi5 in TSB medium. Panel 1 shows results of phage SaMD07phi1 with biofilms grown in wells (1a), on beads (1b) and planktonically (1c). Panel 2 shows results of phage SaRB105030phi5 with biofilms grown in wells (2a), on beads (2b) and planktonically (2c).

After 4 h of treatment with phage SaMD07phi1 in TSB, a 2-log decrease in bacterial quantities (from 5.4 × 10^10^ to 7.7 × 10^8^ CFU/mm^2^) was observed for SaMD07 biofilms grown in 96-well plates (Fig. 3), whereas after 4 h of phage treatment of SaMD07 biofilms grown on beads, no bacteria were recovered in culture (i.e., below the detection limit of 1 × 10^2^ CFU/mm^2^) with controls showing 2.5 × 10^8^ CFU/mm^2^. For biofilms on beads, no significant changes in CFUs were observed at 8 hours however, and a two-log decrease in CFU was observed for biofilms treated with phages (5.0 × 10^6^ CFU/mm^2^) at 24 h as compared to controls (4.7 × 10^8^ CFU/mm^2^). For phage SaRBI05030phi5, SaRBI05030 biofilms grown in 96-well plates did not show a decrease in bacterial counts after 4 h of phage treatment and no difference was observed at 8 and 24 h, while SaRBI05030 biofilms grown on beads showed a decrease in bacterial counts from 1.5 × 10^8^ to 2.0 × 10^7^ CFU/mm^2^ at 4 h, and a decrease from 1.5 × 10^9^ to 5.0 × 10^6^ CFU/mm^2^ at 8 hours. At 24 h, bacterial regrowth was observed but still, phage treated bacterial counts were 1-log lower (1.5 × 10^8^ CFU/mm^2^) that those of the control (2.3 × 10^9^ CFU/mm^2^) for SaRBI05030phi5 and 2-log lower (5.0 × 10^6^ CFU/mm^2^) that those of the control (4.7 × 10^8^ CFU/mm^2^) for SaMD07phi1. Planktonic assay for phage SaMD07phi1 showed 1-log decrease in SaMD07 counts at 4 h and 5-log decrease at 8 hours, and 2-log decrease at 24 h of treatment. Planktonic treatment with SaRBI05030phi5 showed a 2-log decrease in SaRBI05030 at 4 h, an ~ 1-log decrease at 8 hours, and no change at 24 h of treatment. Hence, biofilms grown on beads and tested against phages in TSB medium were suited to susceptibility testing.

### Correlation between bacterial quantification and biolog omnilog

Assay of the same phages using the Biolog Omnilog resulted in hold times of 37 and 28 h for phages SaMD07phi1 and SaRBI05030phi5, respectively, for biofilms grown on beads, and 3 and 2 h, respectively, for biofilms grown in 96-well plates. Correlation between CFU counts and Biolog Omnilog units was linear over time through 8 h of testing (Supplementary Fig. 1). Based on this data, the bead-based assay was selected to determine the activity of eight staphylococcal phages against biofilms formed by 29 bacterial isolates.

### Activity of different phages using the established method

Using the BBTO method, phages SaMD07phi1, SaNSI1469phi1, SaWIQ0493phi1, SaRBI05030phi1, SaRBI03020phi1, Sl46407HNphi1, SaWIQ0488phi1 and SaMD22phi1 were tested against biofilms formed by *S. aureus*, *S. epidermidis*, and *S. lugdunensis* (Table 1). Phage SaWIQ0493phi1 was tested against 18 *S. aureus* isolates; it was highly active against IDRL-6234 biofilms, active against IDRL 6121, IDRL 7542 and IDRL 8457 biofilms, showed intermediate activity against 7 isolates, and was inactive against 7 isolates. Phage SaNSI1469phi1 was tested against 7 *S. aureus* isolates; it was highly active against IDRL-6234 biofilms, active against IDRL 6223, IDRL 6987 and IDRL 8457 biofilms, showed intermediate activity against IDRL-6220 biofilms, and was inactive against two isolates. Phage SaRBI05030phi1 was tested against 6 *S. aureus* isolates; it was active against IDRL 8457 biofilms, showed intermediate activity against IDRL-8960 biofilms, and was inactive against four inactive isolates. Phage SaRBI03020phi1 was tested against 9 *S. aureus* isolates; it was not active against biofilms of any isolate tested. Phage Sl46407HNphi1 was tested against 4 *S. aureus* isolates and was not active against biofilms of any of them. Phage SaWIQ0488phi1 was tested against 15 *S. aureus* isolates; it was highly active against IDRL-6234 biofilms, active against IDRL 6220, IDRL 9646, IDRL 6987, IDRL 7317 and IDRL 8457 biofilms, showed intermediate activity against IDRL-7542 biofilms, and was not active against 8 isolates. Phage SaMD22phi1 was tested against 6 MRSA isolates, against which it showed activity against IDRL-6130, IDRL-6927, IDRL-7381, IDRL-7542 and IDRL-10,947 biofilms, and intermediate activity against IDRL-6987 biofilms. Phage SaMD07phi1 was tested against 29 *Staphylococcus* isolates; it was highly active against IDRL-6220, IDRL-8960, IDRL-6159, and IDRL-10,947 biofilms, active against IDRL-6455, IDRL-6987, IDRL-7317, IDRL-7542, IDRL-8457, IDRL-6108, IDRL-5983, IDRL-6115, IDRL-9756, IDRL6995, IDRL-7530, IDRL-7539, IDRL-7549, and IDRL-9474 biofilms, and inactive against 11 isolates.

**Table 1:** Activity of staphylococcal phages against *Staphylococcus* biofilms.

Phage	Isolate number	Species	Methicillin susceptibility	Planktonic hold time	Biofilm hold time					Activity Category
					Run 1	Run 2	Run 3	Mean	%CV	
SaWIQ0493phi1	IDRL-6116	*S. aureus*	Susceptible	44	0	0	0	0	0	Inactive
	IDRL-6121	*S. aureus*	Susceptible	45	8	8	8	8	0	Active
	IDRL-6220	*S. aureus*	Susceptible	44	6	7	6	6	9.1	Intermediate
	IDRL-6223	*S. aureus*	Susceptible	43	2	7	6	5	52.9	Intermediate
	IDRL-6234	*S. aureus*	Susceptible	44	46	47	47	47	1.2	Highly active
	IDRL-7240	*S. aureus*	Susceptible	44	1	0	0	0	173.2	Inactive
	IDRL-7279	*S. aureus*	Susceptible	15	7	8	6	7	14.3	Intermediate
	IDRL-7303	*S. aureus*	Susceptible	45	0	0	0	0	0	Inactive
	IDRL-8960	*S. aureus*	Susceptible	44	7	7	7	7	0	Intermediate
	IDRL-9321	*S. aureus*	Susceptible	15	0	0	5	2	173.2	Inactive
	IDRL-9646	*S. aureus*	Susceptible	44	6	5	0	4	87.7	Inactive
	IDRL-6987	*S. aureus*	Resistant	41	6	7	7	7	8.7	Intermediate
	IDRL-7317	*S. aureus*	Resistant	21	4	4	7	5	34.6	Intermediate
	IDRL-7542	*S. aureus*	Resistant	11	12	12	9	11	15.7	Active
	IDRL-8457	*S. aureus*	Resistant	20	22	8	9	13	60.1	Active
	IDRL-9057	*S. aureus*	Resistant	45	0	0	0	0	0	Inactive
	IDRL-9756	*S. aureus*	Resistant	41	5	6	7	6	16.7	Intermediate
	IDRL-10,947	*S. aureus*	Resistant	45	0	7	0	2	173.2	Inactive
SaNSI1469phi1	IDRL-6220	*S. aureus*	Susceptible	23	4	7	6	6	27	Intermediate
	IDRL-6223	*S. aureus*	Susceptible	44	8	9	8	8	6.9	Active
	IDRL-6234	*S. aureus*	Susceptible	23	47	47	47	47	0	Highly active
	IDRL-7303	*S. aureus*	Susceptible	15	0	0	0	0	0	Inactive
	IDRL-9321	*S. aureus*	Susceptible	12	0	0	0	0	0	Inactive
	IDRL-6987	*S. aureus*	Resistant	30	8	8	12	9	24.7	Active
	IDRL-8457	*S. aureus*	Resistant	18	9	12	8	10	21.5	Active
SaRB105030phi1	IDRL-7303	*S. aureus*	Susceptible	20	0	0	0	0	0	Inactive
	IDRL-8960	*S. aureus*	Susceptible	44	3	7	7	6	49.9	Intermediate
	IDRL-8457	*S. aureus*	Resistant	21	10	11	13	11	6.2	Active
	IDRL-9057	*S. aureus*	Resistant	27	0	0	0	0	0	Inactive
	IDRL-9756	*S. aureus*	Resistant	41	0	0	0	0	0	Inactive
	IDRL-10,947	*S. aureus*	Resistant	14	0	0	0	0	0	Inactive
SaRB103020phi1	IDRL-6223	*S. aureus*	Susceptible	25	0	0	0	0	0	Inactive
	IDRL-7303	*S. aureus*	Susceptible	15	0	0	0	0	0	Inactive
	IDRL-8960	*S. aureus*	Susceptible	44	0	6	0	3	141.4	Inactive
	IDRL-6987	*S. aureus*	Resistant	22	0	0	0	0	0	Inactive
	IDRL-7542	*S. aureus*	Resistant	28	0	0	0	0	0	Inactive
	IDRL-8457	*S. aureus*	Resistant	20	0	0	0	0	0	Inactive
	IDRL-9057	*S. aureus*	Resistant	15	0	1	0	0	141.4	Inactive
	IDRL-9756	*S. aureus*	Resistant	41	0	0	1	0	0	Inactive
	IDRL-10,947	*S. aureus*	Resistant	18	2	0	1	1	141.4	Inactive
Sl46407HNphi1	IDRL-9646	*S. aureus*	Susceptible	42	0	0	0	0	0	Inactive
	IDRL-8457	*S. aureus*	Resistant	22	0	0	0	0	0	Inactive
	IDRL-9756	*S. aureus*	Resistant	44	0	1	0	0	212.1	Inactive
	IDRL-10,947	*S. aureus*	Resistant	42	0	0	0	0	0	Inactive
SaWIQ0488Phi1	IDRL-6116	*S. aureus*	Susceptible	44	0	0	1	0	0	Inactive
	IDRL-6220	*S. aureus*	Susceptible	44	8	8	8	8	0	Active
	IDRL-6223	*S. aureus*	Susceptible	43	0	0	0	0	0	Inactive
	IDRL-6234	*S. aureus*	Susceptible	44	47	48	47	47	1.5	Highly Active
	IDRL-7303	*S. aureus*	Susceptible	26	0	0	0	0	0	Inactive
	IDRL-8960	*S. aureus*	Susceptible	44	0	0	0	0	0	Inactive
	IDRL-9321	*S. aureus*	Susceptible	44	0	0	0	0	0	Inactive
	IDRL-9646	*S. aureus*	Susceptible	44	8	8	8	8	0	Active
	IDRL-6987	*S. aureus*	Resistant	16	8	8	8	8	0	Active
	IDRL-7317	*S. aureus*	Resistant	44	8	8	8	8	0	Active
	IDRL-7542	*S. aureus*	Resistant	14	6	6	5	6	0	Intermediate
	IDRL-8457	*S. aureus*	Resistant	20	10	19	10	15	43.9	Active
	IDRL-9057	*S. aureus*	Resistant	11	0	0	0	0	0	Inactive
	IDRL-9756	*S. aureus*	Resistant	41	0	0	0	0	0	Inactive
	IDRL-10,947	*S. aureus*	Resistant	45	0	0	0	0	0	Inactive
SaMD07phi1	IDRL-6121	*S. aureus*	Susceptible	45	0	0	0	0	0	Inactive
	IDRL-6152	*S. aureus*	Susceptible	19	0	0	0	0	0	Inactive
	IDRL-6175	*S. aureus*	Susceptible	46	0	1	0	0	57.7	Inactive
	IDRL-6220	*S. aureus*	Susceptible	44	48	48	41	46	2.9	Highly active
	IDRL-6223	*S. aureus*	Susceptible	43	0	0	0	0	0	Inactive
	IDRL-6234	*S. aureus*	Susceptible	44	0	0	0	0	0	Inactive
	IDRL-6455	*S. aureus*	Susceptible	48	9	9	8	9	2.2	Active
	IDRL-7240	*S. aureus*	Susceptible	44	0	0	0	0	0	Inactive
	IDRL-7279	*S. aureus*	Susceptible	13	0	0	0	0	0	Inactive
	IDRL-7303	*S. aureus*	Susceptible	45	0	0	0	0	0	Inactive
	IDRL-8960	*S. aureus*	Susceptible	44	47	48	15	37	17.1	Highly active
	IDRL-9321	*S. aureus*	Susceptible	44	0	0	0	0	0	Inactive
	IDRL-9646	*S. aureus*	Susceptible	44	0	0	0	0	0	Inactive
	IDRL-6159	*S. aureus*	Resistant	43	47	47	12	35	19.1	Highly active
	IDRL-6987	*S. aureus*	Resistant	45	48	18	0	22	36.7	Active
	IDRL-7317	*S. aureus*	Resistant	44	15	19	0	11	29.5	Active
	IDRL-7542	*S. aureus*	Resistant	44	47	17	0	21	37.2	Active
	IDRL-8457	*S. aureus*	Resistant	19	44	0	0	15	57.7	Active
	IDRL-9057	*S. aureus*	Resistant	45	0	0	0	0	0	Inactive
	IDRL-9756	*S. aureus*	Resistant	41	45	47	0	31	28.9	Active
	IDRL-10,947	*S. aureus*	Resistant	45	47	48	24	40	11.4	Highly active
	IDRL-6108	*S. epidermidis*	Susceptible	46	46	18	1	22	35	Active
	IDRL-5983	*S. lugdunensis*	Resistant	43	12	10	8	10	6.7	Active
	IDRL-6115	*S. aureus*	Susceptible	43	13	12	11	12	2.8	Active
	IDRL-6995	*S. lugdunensis*	Susceptible	17	18	13	11	14	8.6	Active
	IDRL-7530	*S. lugdunensis*	Susceptible	37	16	14	0	10	29.1	Active
	IDRL-7539	*S. lugdunensis*	Susceptible	34	31	11	0	14	37.4	Active
	IDRL-7549	*S. lugdunensis*	Resistant	40	17	11	8	12	12.7	Active
	IDRL-9474	*S. lugdunensis*	Susceptible	40	18	18	10	15	10	Active
SaMD22Phi1	IDRL-6130	*S. aureus*	Resistant	17	8	10	9	9	11.1	Active
	IDRL-6927	*S. aureus*	Resistant	15	9	8	8	8	6.9	Active
	IDRL-6987	*S. aureus*	Resistant	45	7	6	9	7	20.8	Intermediate
	IDRL-7381	*S. aureus*	Resistant	46	6	12	7	8	38.6	Active
	IDRL-7542	*S. aureus*	Resistant	44	13	7	13	11	31.5	Active
	IDRL-10,947	*S. aureus*	Resistant	45	6	9	9	8	21.7	Active

*%CV* Percent coefficient of variance.

Among all the isolates tested, high activity of phages SaWIQ0493phi1, SaNSI1469phi1, and SaWIQ0488phi1, with mean hold times 47, 47, and 48 h, respectively, was shown against IDRL-6234 biofilms. Activity of phages SaWIQ0493phi1, SaNSI1469phi1, SaRBI05030phi1, SaWIQ0488phi1, and SaMD07phi1 was shown against MRSA IDRL-8457 biofilms. Activity of phages SaNSI1469phi1, SaWIQ0488phi1, and SaMD07phi1 was shown against MRSA isolate IDRL-6987 biofilms. Activity of phages SaWIQ0493phi1, SaMD07phi1, and SaMD22phi1 was shown against MRSA IDRL-7542 biofilms. Overall, considering the 43 MRSA-phage combinations tested, 47% (20/43) of phages showed activity against MRSA biofilms.

### Reproducibility of biofilm phage susceptibility testing

**Fig. 4:**
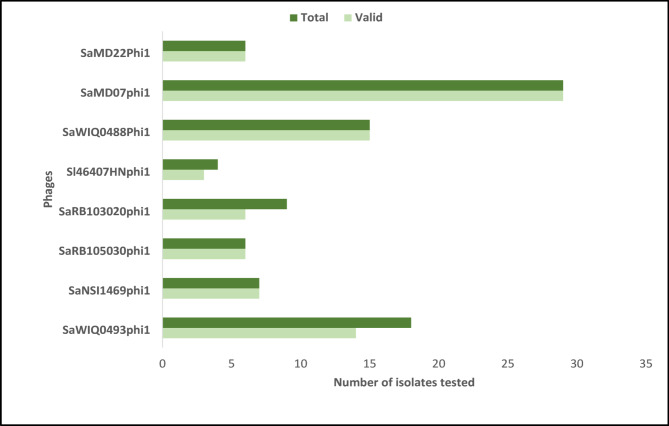
Reproducibility of biofilm phage susceptibility testing of 8 phages against 29 isolates.

The reproducibility of the biofilm phage susceptibility testing was considered valid if the percent coefficient of variance was ≤ 75% for three results generated on three separate days. Phage SaWIQ0493phi1 resulted in 78%, phage SaRBI05020phi1 in 67%, phage Sl46407HNphi1 in 75%, and phages SaNSI1469phi1, SaRBI05030phi1, SaWIQ0488Phi1, SaMD07phi1 and SaMD22phi1 in 100% valid reproducible biofilm susceptibility tests across three replicates on three different days (Fig. 4). Isolates IDRL-6987, IDRL-7317, IDRL-7542, IDRL-9756, IDRL-6108, IDRL-7530 and IDRL-7539 exhibited high hold times across two runs, and inactivity in the third run. Isolate IDRL-8457 exhibited a hold time of 44 h in first run, with inactivity in the two other runs. These discrepant phages in third run were outliers. Phage-bacteria pairs that resulted in invalid (high %CV) classification was a result of variable dyads in hold times. In total, biofilm susceptibility assessment of 8 phages with 29 isolates resulted in 91% of phage-biofilm assays performed in three replicates on three different days classified as valid.

## Discussion

Phages have potential as biological tools to manage bacterial biofilm-associated infections. Yet, there remains limited understanding of factors that impact the effectiveness of phages against biofilms. Experimental variables such as biofilm growth conditions may affect antibiofilm activity^16^. Considering the diversity in methodologies utilized, standardized in vitro approaches to characterize phage/biofilm interactions and assess potential phage activity are required.

The present study compared in vitro experimental conditions for biofilm growth in 96-well plates and on glass beads, phage-biofilm assay conditions using PBS and TSB medium and two biofilm quantification methods – bacterial reduction of viable colonies and a metabolic assay -, using two model phages. Biofilms should undergo testing in environments that mirror conditions of their superhosts; culture media abundant in nutrients may differ from bodily fluids or environments where biofilms develop in animals. PBS (phosphate-buffered saline) was initially selected as a low-nutrient substitute. Unfortunately, PBS did not support biofilm growth or biofilm reduction by phages, so TSB was selected. A prior study supports use of nutrient-rich media for testing biofilm susceptibility to phage^16^. Given the reliance of phages on their hosts, changes in bacterial metabolic status may impact phage infection dynamics and viral replication. The availability of nutrients may also impact bacterial physiology. Hence, nutrient rich media allow bacterial hosts to flourish and help phages in infection dynamics^9,18^.

Biofilms of bacterial hosts SaMD07 and SaRBI05030 grown in wells of microtiter well plates and on glass beads in 96-well plates were compared. Biofilms grown in wells of microtiter plates showed 2-log higher bacterial quantities compared to those grown on glass beads. The same titer of phages (4 × 10^8^ PFU/ml) was tested against these biofilms, affecting the phage multiplicity of infection (MOI). Notably, there were improved biofilm reductions on glass beads compared to microtiter well plates, with two reduction patterns observed. Phage SaMD07phi1 resulted in no culturable bacterial amounts on beads at 4 h, although an increase in bacteria was noted at 8 hours followed by a decrease at 24 h. In contrast, with phage SaRBI05030phi5, only a 1-log decrease in biofilms on beads was observed at 4 h, followed by a 3-log decrease at 8 hours and an increase at 24 h. Dynamics of biofilm changes can be specific to phages and their specific hosts. Phages displaying larger burst sizes and shorter latent periods may be optimal for targeting biofilms^16^. The two patterns of biofilm activity observed have been studied by Simmons and colleagues who report evolutionary oscillations in phage and bacteria dynamics in biofilm infections^19^. This scenario is also observed in antibiotic susceptibility testing of biofilms, where biofilm persister cells become prominent under antibiotic pressure but not so much without it^20^.

Evaluation methods for biofilm quantification rely on diverse parameters, potentially yielding disparate outcomes when used individually to evaluate phage control of biofilms. To assess phage antibiofilm activity, a comparison among two bacterial quantification assessment methods - CFU quantification and metabolic activity - was conducted, with a linear relationship between CFU and Omnilog units observed over time.

Using the bead method, 8 staphylococcal phages were tested against biofilms of 29 isolates of *S. aureus*, *S. epidermidis* or *S. lugdunensis*. Hold times varied. More than one phage was active against biofilms of IDRL- 6234, IDRL-8457, IDRL-6987 and IDRL-7542. Infection of a single bacterium by multiple phages suggests the presence of either same or multiple phage receptors on its surface^21^. Isolate IDRL-6234 exhibited high hold times with three phages - SaWIQ0493phi1, SaNSI1469phi1 and SaWIQ0488phi1, which may suggest that these isolates are weak biofilm formers^22^have fewer persister cells in their biofilms or supports a short latent period and higher phage burst size. Given that only one MOI was tested, phages with inactive or intermediate activity could potentially show better activity if higher titers^23^longer treatment periods^16^phage cocktails^24^ or phage lysins were to be used, or if phages had been applied in combination with antibiotics^9,25^. Resistance to phage lysins is less than to whole phages. Numerous mechanisms contribute to the effectiveness of phage infection in genetically and physiologically identical hosts on the transcriptomic and proteomic level^26^. Considering that biofilm formation architecture varies under identical growth conditions, the biofilm susceptibility testing exhibited good reproducibility over three different days. However, challenges with reproducibility have been encountered in the assessment of planktonic phage susceptibility testing^17^ and antibiotic susceptibility of biofilms^27^.

In conclusion, this study underscores the importance of optimizing experimental conditions for biofilm phage susceptibility testing to achieve reliable and reproducible results. By systematically comparing biofilm growth platforms, media compositions, and quantification methods, glass beads were identified as an effective platform for biofilm growth and TSB as a suitable medium for supporting bacterial and phage activity. The study also demonstrated the linear correlation among CFU and metabolic activity assessments of phage-exposed biofilms. The BBTO method exhibited good reproducibility for assessing staphylococcal phage activity tested against *Staphylococcus* biofilms.

Overall, this research advances standardization of in vitro methodologies for phage susceptibility testing, laying the groundwork for evaluation of phages as therapeutic agents for managing biofilm-associated infections. Future studies should explore diverse phage families, varying phage titers, phage cocktails, and phage-antibiotic combinations.

## Data Availability

All data supporting the findings of this study are available within the manuscript.
